# Assessing myBaits Target Capture Sequencing Methodology Using Short-Read Sequencing for Variant Detection in Oat Genomics and Breeding

**DOI:** 10.3390/genes15060700

**Published:** 2024-05-27

**Authors:** Khalid Mahmood, Pernille Sarup, Lukas Oertelt, Ahmed Jahoor, Jihad Orabi

**Affiliations:** 1 Nordic Seed, Grindsnabevej 25, 8300 Odder, Denmark; pesa@nordicseed.com (P.S.); ahja@nordicseed.com (A.J.); jior@nordicseed.com (J.O.); 2 Nordic Seed Germany, Kirchhorster Str. 16, 31688 Nienstädt, Germany; luoe@nordicseed.com

**Keywords:** oat genome, myBaits technology, targeted sequencing, variant calling, genetic variants, genomic regions

## Abstract

The integration of target capture systems with next-generation sequencing has emerged as an efficient tool for exploring specific genetic regions with a high resolution and facilitating the rapid discovery of novel alleles. Despite these advancements, the application of targeted sequencing methodologies, such as the myBaits technology, in polyploid oat species remains relatively unexplored. In this study, we utilized the myBaits target capture method offered by Daicel Arbor Biosciences to detect variants and assess their reliability for variant detection in oat genomics and breeding. Ten oat genotypes were carefully chosen for targeted sequencing, focusing on specific regions on chromosome 2A to detect variants. The selected region harbors 98 genes. Precisely designed baits targeting the genes within these regions were employed for the target capture sequencing. We employed various mappers and variant callers to identify variants. After the identification of variants, we focused on the variants identified via all variants callers to assess the applicability of the myBaits sequencing methodology in oat breeding. In our efforts to validate the identified variants, we focused on two SNPs, one deletion and one insertion identified via all variant callers in the genotypes KF-318 and NOS 819111-70 but absent in the remaining eight genotypes. The Sanger sequencing of targeted SNPs failed to reproduce target capture data obtained through the myBaits technology. Similarly, the validation of deletion and insertion variants via high-resolution melting (HRM) curve analysis also failed to reproduce target capture data, again suggesting limitations in the reliability of the myBaits target capture sequencing using short-read sequencing for variant detection in the oat genome. This study shed light on the importance of exercising caution when employing the myBaits target capture strategy for variant detection in oats. This study provides valuable insights for breeders seeking to advance oat breeding efforts and marker development using myBaits target capture sequencing, emphasizing the significance of methodological sequencing considerations in oat genomics research.

## 1. Introduction

The cultivated oat (*Avena sativa* L.) genome has recently been sequenced, providing valuable insights into this healthy cereal crop [1,2]. Oats are recognized for their importance as a source of carbohydrates, dietary soluble fiber, balanced protein, lipids, phenolic compounds, vitamins, and minerals, rendering them a promising functional food with diverse health benefits [3]. The oat genome is an allohexaploid (AACCDD, 2n = 6× = 42) with six sets of chromosomes [1,2,4]. Its genome complexity, due to its hexaploid nature and mosaic-like architecture, has offered challenges in studies for research and breeding [1,2,4]. The sequencing of the oat genome has significant implications for both agriculture and human nutrition. In agriculture, it provides greater knowledge of oat genomics, offering more opportunities for targeted improvements in yield, disease tolerance, and other characteristics of oats [1,2,3,4]. In human nutrition, oats are esteemed as a valuable source of nutrients, and they have been associated with various health benefits, including the mitigation of cardiovascular disease risks, inflammation, and type-2 diabetes [3,5,6].

The complexity of the oat genome emphasizes the indispensability of targeted sequencing in studying this crop [1,2]. Despite remarkable advances in sequencing technologies and bioinformatics techniques in recent years, conducting genome sequencing on a large scale to a sufficient depth remains challenging for plants with large and highly repetitive genomes like oats. Target capture based on hybridization offers a cost-effective means of attaining high depth coverage and identifying sequence variants in the coding and noncoding regions of very large genomes [7,8,9,10]. This approach involves a custom design of capture probes targeting specific chromosome regions harboring loci or candidate genes for traits of interest, enabling the highly flexible scaling of resequencing experiments from a few to many genes at a low cost for large plant populations [7,9]. Targeted gene enrichment utilizes synthetic DNA probes designed from reference sequences that are complementary to specific regions in genomes. These probes are attached to a substrate to facilitate the capture of targeted DNA regions. Subsequently, the captured DNA can undergo high-throughput sequencing without requiring universal primers [11]. This technique is widely employed in human genomic research, phylogenetic studies, and evolutionary investigations [12,13]. Surprisingly, gene enrichment has not yet been explored in oat breeding and research. Several factors contribute to this; for example, (1) Oats have a complex polyploid genome, and the presence of multiple sets of chromosomes and extensive repetitive regions complicates the situation [14]. This complexity makes it difficult to design effective baits for target capture and accurately identify genetic variants. (2) Developing and optimizing gene enrichment techniques require substantial technical expertise and financial investment. Oat research programs, often less funded compared to major crops like wheat or barley, may lack the resources needed to implement and refine these advanced methodologies. (3) Oat breeding has traditionally relied on conventional methods. The integration of molecular techniques, including gene enrichment and targeted sequencing, has been slower due to the established reliance on these conventional approaches. (4) Research priorities and funding are often directed towards crops with higher economic importance or those considered staple foods. As a result, oats, which are important but not among the top global crops, have seen less investment in advanced genomic technologies. (5) The last and most important factor is that, until recently, there was a lack of oat genomic resources. The lack of a high-quality reference genome and the limited availability of annotated gene sequences have hindered the development and application of targeted sequencing technologies. Recent advancements in oat genomics are beginning to address these gaps.

In this context, the myBaits technology, a hybridization capture system, has been used for the targeted next-generation sequencing of specific genomic regions of interest, providing a powerful and versatile tool for studying the genome [9,10,11]. The myBaits technology provides targeted sequencing solutions for plant genomics. These kits use hybridization capture with biotinylated RNA baits to enrich specific genomic regions efficiently, providing deep insights into plant genomes [10,15]. Compared to traditional shotgun techniques, the myBaits technology enables next-generation sequencing (NGS) to be an order of magnitude more efficient by enriching target molecules and removing non-target molecules, resulting in significant cost savings compared to shotgun sequencing approaches [8,12,13]. Additionally, the myBaits Custom DNA-Seq kits are versatile and can accommodate various sample types like genomic DNA, metagenomic DNA, environmental DNA, ancient DNA, and more, making them ideal for gene or exon resequencing, novel variant discovery, phylogenetics, transgene detection, and other research applications in plant genomics [2,3,4,8,12,13]. When dealing with high coverage of short sequence reads from specific regions of a crop genome, the initial step involves aligning these reads to corresponding regions of a reference genome. Various mapping tools employ distinct algorithms to ensure the precise and efficient alignment of these short-sequence reads to the appropriate locations on the reference genome [16,17]. Polyploid crop genomes, such as oats, significantly amplify the complexity and challenges associated with both sequence mapping and variant detection. Therefore, using the right algorithm becomes imperative to ensure precision, accuracy, and reproducibility. In our study, we employed various variant-calling tools and focused on variants detected via all of them to enhance the reliability of our results. Therefore, we focused on the common variants to ensure the accuracy and reliability of the oat target capture data generated in this study.

The objective of this study was to evaluate the efficacy and reliability of the myBaits target capture sequencing technology for variant detection in oat genomics. Specifically, the study aimed to utilize the myBaits technology using short-read sequencing to detect variants in specific regions of the oat genome and assess the reliability of identified variants through rigorous validation efforts.

## 2. Materials and Methods

### 2.1. Plant Material for DNA Extraction

Ten oat genotypes were carefully selected for target capture using short-read sequencing. The oat genotypes were chosen to represent a wide range of genetic diversity, local adaptability, and breeding relevance. This diversity is essential in identifying a wide array of genetic variants and assessing the efficacy of the myBaits technology across different oat lines. These genotypes were Symphony, Delfin, NOS 81920-15, KF-318, Mathilda, WPB_Oskar, NOS 81937-11, NOS 81950-13, NOS 819111-70, and NOS 819111-120. The plant material was germinated in the controlled environment of greenhouse facilities of Nordic Seed A/S, ensuring optimal conditions of temperature and light. Seedlings were kept under 16 h of daylight at 18–24 °C and 8 h of darkness at 14–16 °C. After seven days, the lower sections of two coleoptiles and primary leaves were carefully excised and preserved in a 96-well Micro-Dilution Tube System (STARLAB International GmbH, Hamburg, Germany) containing glass beads. These plant tissue samples were then stored at −20 °C for two days before undergoing a two-day freeze-drying process. DNA extraction followed an adapted SDS-based method outlined by Pallotta et al. [18]. The quality of the extracted DNA was assessed by measuring its concentration and 260/280 nm absorption ratio using an Epoch ™ microplate spectrophotometer (Biotek^®^ Instruments, Winooski, VT, USA), while DNA integrity was evaluated through size separation on a 1.2% (*w*/*v*) agarose gel.

### 2.2. Bait Design and Target Capture Sequencing

We targeted a genomic region situated on chromosome 2A, spanning positions 453,601,785 to 456,853,474, which encompasses 98 annotated genes within the Sang cv genome [1]. A comparative genomic analysis against the PepsiCo reference genome (https://wheat.pw.usda.gov/jb?data=/ggds/oat-ot3098v2-pepsico, accessed on 5 April 2024) unveiled a significant match localized on the 7D chromosome, ranging from positions 453,972,539 to 457,233,851. To enable precise targeted capture sequencing, we initially processed target sequences with 268,722 nucleotides of the 98 genes within the region. For target capture sequencing, we designed the baits for the myBaits hybridization step, utilizing 80 nt probes with a 4× tiling strategy, effectively placing a probe approximately every ~20 nt along the target region.

All designed baits underwent rigorous scrutiny through BLAST analysis against three genomes (Oat_OT3098_v2.dna.toplevel.fas.gz, Asativa_sang.V1.1.dna.toplevel.fa.gz, GCA_023646675.1_ASM2364667v1_genomic.fna.gz), including a plastid genome (plastid NC_027468.1). In adherence to filtration criteria, 7296 baits met the stringent parameters, exhibiting less than or equal to 25% softmasking for repeats and no hits to the plastid genome. Conversely, the Relaxed Design Option accommodated all baits that adhered to more lenient filtration criteria, maintaining softmasking of less than or equal to 35% for repeats and no hits to the plastid genome (Bait design Excel file and Supplementary Materials). We selected the baits that met the stringent parameter for target enrichment.

Subsequently, sequencing services were outsourced to Daicel Arbor Bioscience, Ann Arbor, MI, USA, wherein 10 samples were subjected to sequencing utilizing NovaSeq with PE150. This process yielded a cumulative total of 34 Gbp of data, with an average of 3.4 Gbp per sample across the 10 samples. The sequencing effort was facilitated through the procurement of requisite materials, including the following kits: (a) myReads Standard DNA Package for 1–24 samples, (b) myBaits Custom 1–20 K Reorder 16 Rxn, and myReads NovaSeq S4 service for PE150. All the resulting sequencing data was deposited in NCBI with accession number 1095189, and they are accessible via https://dataview.ncbi.nlm.nih.gov/object/PRJNA1095189?reviewer=74ca1vatg0jmg1fmvql5jlj33o, accessed on 10 April 2024.

### 2.3. Mapping and Variant Calling

For read mapping, we employed three different read aligners, BWA MEM v.0.7.17 [19], Bowtie2 v.2.3.5.1 [20], and NGSEP v.4.1.0 [21] mappers, which were used for read alignment when using the Sang cv oat reference genome sequence. The alignment using the Bowtie 2 and BWA-MEM was performed using the Curiogenomic platform (https://www.curiogenomics.com, accessed on 23 March 2024). The read mapping using the NGSEP was performed using the genome DK cluster (https://genome.au.dk/, accessed on 1 April 2024). We implemented several quality-control measures before and during the read-mapping and variant-calling processes, such as pre-mapping quality checks, trimming, mapping quality assessment, variant filtration and multi-caller validation, and manual inspection. During the pre-mapping quality checks, we performed quality checks using FastQC to assess the quality of the raw sequencing data. FastQC provided detailed information on various quality metrics, including base quality scores, GC content, and sequence duplication levels. Sequencing adapters and low-quality bases were then trimmed from the raw reads using Trimmomatic. Parameters were set to remove leading and trailing bases below a quality threshold of 20 and to trim reads when the average quality within a four-base sliding window dropped below 20. Reads shorter than 150 bases after trimming were discarded.

Variant calling was executed utilizing five distinct tools incorporating FreeBayes v. 1.3.1 [22], GATK HaplotypeCaller (HC) v. 4.2.3 [23], SAMtools-mpileup (version 1.9) [24] and DeepVariant v. 1.2.0 [25], and NGSEP with default settings. In the cases of GATK haplotypeCaller, FreeBayes, and DeepVariants, variants were called using BWA-MEM alignments, and in the case of SAMtools-mpileup, variants were called using Bowtie2 alignments. In the case of NGSEP variant calling, we used the NGSEP-based alignment. All the variant callers were implemented with the default settings and variants filtered based on sequencing quality (QUAL < 30 and minimum read coverage of 5) and other recommended parameters using respective variant caller guidelines. All the variants were used at default settings and focused on variants identified via all five callers. This cross-caller consensus approach minimized false positives and ensured that only high-confidence variants were considered for validation. In the end, we performed a manual inspection using the Integrative Genomics Viewer (IGV) to visually confirm variant calls and assess read alignments. Ultimately, the functional annotations of the variants were predicted via snpEff (version 4.3).

### 2.4. Validation of Targeted Variants

The validation process was initiated by selecting two SNP variants (2A_456055130 and 2A_455932982), along with one deletion variant and one insertion variant found in genotypes KF-318 and NOS 819111-70 but not in the other eight genotypes. For validation in the case of SNP variants, PCR amplification and Sanger sequencing were conducted, whereas deletion and insertion variants were validated through high-resolution melting curve analysis (HRM). Primers flanking the target variants were designed and synthesized (Supplementary Table S1), followed by PCR using genomic DNA from oat genotypes KF-318 and NOS 819111-70. The PCR products underwent purification and sequencing using Sanger technology. The Sanger sequencing data were analyzed using Geneious Prime (https://www.geneious.com, accessed on 23 March 2024) and to determine the nucleotide sequences around the selected variants. Heterozygosity or homozygosity for the chosen SNPs was identified by comparing the sequencing results with the reference genome sequence using Geneious Prime (https://www.geneious.com, accessed on 23 March 2024). In the case of deletion and insertion variants, Sanger sequencing was not conducted; instead, validation was achieved through high-resolution melting (HRM) curve analysis. The clustering of genotypes based on HRM curve analysis was used to confirm the presence or absence of the variants. The HRM curve patterns of genotypes containing the validated variants were compared with those lacking these variants. The combined interpretation of results from PCR amplification, Sanger sequencing, and HRM curve analysis facilitated the validation of the selected variants.

## 3. Results

Three different aligners, BWA-MEM, Bowtie2, and NGSEP, were evaluated using Illumina paired-end read target capture datasets from the 10 oat genotypes. The results of the read statistics and mapping efficiency analysis across the ten oat genotypes using BWA-MEM, Bowtie 2, and NGSEP aligners are summarized in Table 1. The table presents the total number of reads generated for each genotype, the reads that passed quality-control filtering, and the successfully mapped reads via each aligner—BWA-MEM, Bowtie 2, and NGSEP. BWA-MEM consistently demonstrates high mapping efficiency across all genotypes, with percentages ranging from 98.98% to 99.84%. Bowtie 2 and NGSEP also showed satisfactory mapping efficiencies, albeit with slight variations across genotypes. This provides insights into aligner performance, aiding in the selection of the most suitable tool for subsequent oat genomics analyses. The implications of variations in read-mapping efficiency are significant for accurate variant detection in the complex oat genome. High mapping efficiency indicates robust performance in handling the repetitive regions and polyploid nature of oats, which is essential for reliable variant calling. However, variations in mapping efficiency observed with different mappers suggest potential challenges in aligning reads in certain genotypes, which could introduce biases and inaccuracies in downstream analyses.

The variant-calling results were obtained from different variant callers across a range of oat genotypes (Table 2). The GATK Haplotype Caller (GATK HC) detected varying variant counts, from 3816 for Symphony to 4411 for NOS 819111-120. In contrast, SAMtools mpileup identified fewer variants compared to GATK HC, ranging from 753 for Symphony to 2820 for NOS 819111-120. FreeBayes demonstrated a broad spectrum of variants across genotypes, with counts ranging from 544 for NOS 81937-11 to 4338 for NOS 81920-15. DeepVariant identified fewer variants in the majority of genotypes compared to all variant callers and exhibited variant counts ranging from 513 for NOS 81937-11 to 2185 for Symphony. Lastly, the NGSEP Variant caller consistently detected variants across genotypes, ranging from 3223 for Symphony to 4325 for NOS 819111-120. The observed variations in variant calling across different aligners indicate the importance of interpreting the accuracy and confidence of the identified genetic variants. Variants identified exclusively via one caller and not via others are more likely to be false positives, highlighting the need for a consensus approach in variant detection. This is one of the reasons why we took the cross-caller consensus approach to minimize the false positives and ensure that only high-confidence variants were considered. Moreover, we selected the variants for validation that were present in the two target genotypes and absent in the remaining genotypes. This was done with the aim of increasing the stringency and reliability of variant detection.

**Figure 1:**
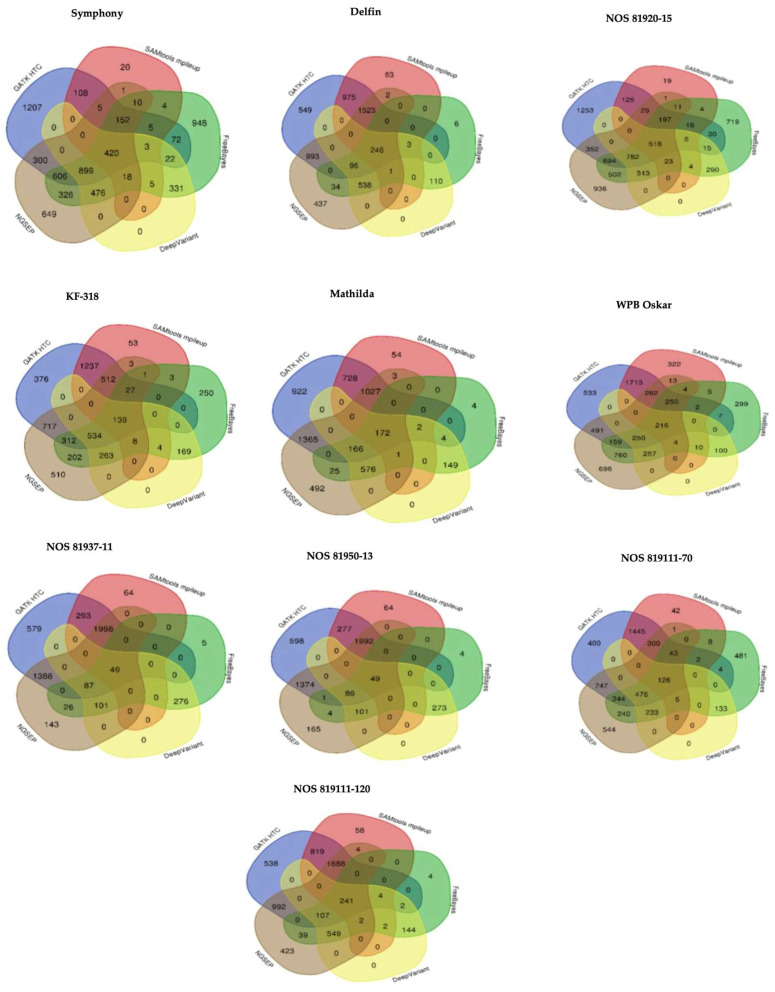
Comparison of identified variants using different variant callers for each genotype. Among all the variant callers, DeepVariant identified variants that were also identified via one of the other variant callers in all the genotypes.

Upon comparing genotypes, we found 420 variants identified via all variant callers in the Symphony genotype. Specifically, GATK HC exclusively identified 1207 variants, FreeBayes identified 948 variants not identified via any other caller, NGSEP identified 649 unique variants, and Samtools mpileup detected 20 variants not found via any other caller (Figure 1). These variants were considered false positives if they were only identified via one caller and absent when other callers were used. Regarding DeepVariant, all the variants identified were also detected via at least one other caller across all the investigated genotypes. For the Delfin genotype, 246 variants were detected via all callers, of which 549 were uniquely identified via GATK HTC, 437 via NGSEP, 6 via FreeBayes, and 63 via Samtools mpileup (Figure 1). For the 81920-15 genotype, 518 variants were detected via all callers, with 1253 variants uniquely identified via GATK HC, 936 identified via NGSEP, 719 identified via FreeBayes, and Samtools mpileup detecting 19 variants. Similar patterns were observed for other genotypes, as depicted in Figure 1. All the variants identified via DeepVariant across all genotypes were also identified via one or more other callers, with none uniquely identified via DeepVariant. These results emphasize that DeepVariant does not produce false positives and provides more reliable variant detection. These results provide insights into the performance and efficacy of different variant callers in identifying genetic variants within oat genotypes.

### Results of the Validation of Targeted Variants

**Figure 2:**
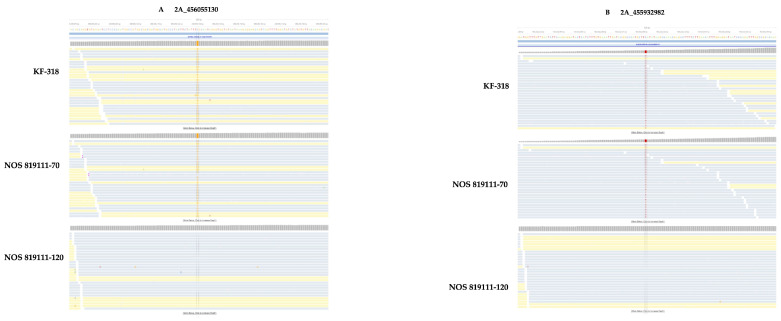
Read alignment of target capture sequencing data of oat genotypes KF-318, NOS 819111-70, and NOS 819111-120 flanking the selected SNPs on chromosome 2A at positions 456055130 and 455932982. (**A**) Read alignment of the SNP 2A_456055130 and nucleotide letter highlighted in orange (G) represented the SNP in KF-318 and NOS 819111-70. (**B)** Read alignment of the SNP 2A_455932982 and nucleotide letter highlighted in red (T) represented the SNP in KF-318 and NOS 819111-70 Above read alignment obtained through BWA-MEM mappers and genotype NOS 819111-120 used as example for remaining seven oat genotypes that exhibit similar pattern as that observed in NOS 819111-120. For SNP 2A_455932982, we did not have coverage of this SNP in the target capture data for two genotypes (Symphony and NOS 81920-15); hence, NOS 819111-120 used as example for remaining five oat genotypes.

To validate our findings regarding the variants identified via all variant callers, we selected two SNP variants, one deletion variant, and one insertion variant present in genotypes KF-318 and NOS 819111-70 but absent in the remaining eight genotypes (Supplementary Figures S1–S4). We performed PCR amplification and Sanger sequencing to confirm the presence of the selected SNPs (2A_456055130 and 2A_455932982), which was consistently identified via all variant callers in genotypes KF-318 and NOS 819111-70. These SNPs are located on chromosome 2A at positions 456055130 and 455932982, respectively. In the target capture sequencing data, SNP 2A_456055130 appeared as a G in both KF-318 and NOS 819111-70, while it was a C in the remaining genotypes and the reference (Figure 2A). The total depth coverage for different genotypes ranged from 70 to 450, with a total depth coverage of 380 (540 reads) and 350 in KF-318 and NOS 819111-70, respectively. The genotype 819111-70 has a total coverage of 637 (906 reads) for this SNP. We did not observe this SNP in all the genotypes except in KF-318 and NOS 819111-70 (Supplementary Figure S1). Similarly, SNP 2A_455932982 was identified as a T in both genotypes and a C in the reference and other genotypes, with total depth coverage of 171 In KF-318 (308 reads) and 141 (257 reads) in 819111-70 (Figure 2A). On the other hand, the NOS 819111-120 genotype has a total depth coverage of 525 (947 reads) for this SNP and does not exhibit any heterozygous allele. We did not observe this SNP in the six genotypes except KF-318 and NOS 819111-70 (Supplementary Figure S2), and two genotypes (Symphony and 81920-15) did not exhibit coverage of this SNP. No heterozygosity of these SNPs in KF-318 and NOS 819111-70 was observed in the target capture data for both variants either (Figure 2A,B).

**Figure 3:**
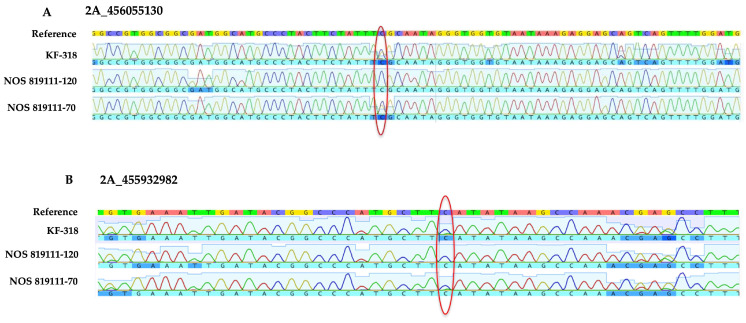
Sanger sequencing flanking selected SNPs (2A_456055130 2A_455932982). Alignment of KF-318 clearly shows heterozygous SNP in KF-318 and NOS 819111-70 but is absent in NOS 819111-120 (**A**). Similarly, results of amplification and alignment shown for SNP 2A_455932982 (**B**), for which we do not see any polymorphism. The red circle pinpoint the position of selected SNPs in different genotypes.

The Sanger sequencing of the PCR products flanking these SNPs revealed heterozygosity for SNP 2A_456055130 in both KF-318 and NOS 819111-70 (Figure 3A), contradicting the target capture data. Similarly, for SNP 2A_455932982, only a “C” nucleotide was observed in both genotypes, contrary to the target capture data (Figure 3B). Although a faint “T” was observed in KF-318 and NOS 819111-70, it was deemed unreliable. Even if it were considered genuine, it still contradicted the target capture data, where no instances of “C” were observed at this specific location in these two genotypes.

**Figure 4:**
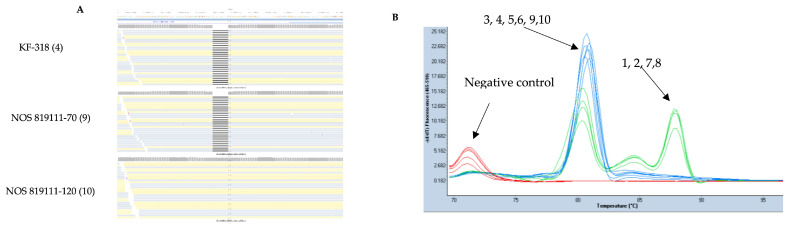
Read alignment highlighting 9 bp deletion in genotypes KF-318 and NOS 819111-70 (**A**) Absence of 9 bp deletion in genotype 819111-120 on chromosome 2A at positions 453603957 and depicted using black line. High-resolution melting (HRM) curve chromatogram of all 10 oat genotypes (**B**). KF-318, NOS 819111-70, and NOS 819111-120 cluster together in HRM. Genotypes are represented as follows: (1) Symphony, (2) Delfin, (3) NOS 81920-15, (4) KF-318, (5) Mathilda, (6) WPB Oskar, (7) NOS 81937-11, (8) NOS 81950-13, (9) NOS 819111-70, and (10) NOS 819111-120. Read alignment obtained through BWA-MEM mappers and genotype NOS 819111-120 used as example for remaining seven oat genotypes that exhibit similar alignment pattern as observed in NOS 819111-120. Alignment for all genotypes provided in Supplementary Figure S3.

Using genotypes KF-318 and NOS 819111-70 as references, we identified deletions and insertions compared to other genotypes. One deletion variant (9 bp) and one insertion variant (3 bp) were selected. The 9 bp deletion, located on chromosome 2A at position 453603957, was clearly identified in KF-318 and NOS 819111-70 (Figure 4A), with a total depth coverage of 420 (562 reads) and 442 (591 reads), respectively. The genotype 819111-120 has coverage of 620 (830 reads) for this region. We did not observe this 9bp deletion in all the genotypes except in KF-318 and NOS 819111-70 (Supplementary Figure S3). High-resolution melting (HRM) curve analysis grouped the ten oat genotypes into two clusters (Figure 4B), with genotypes KF-318 and NOS 819111-70 clustering together, along with those that did not contain the 9 bp deletion (e.g., NOS 819111-120).

**Figure 5:**
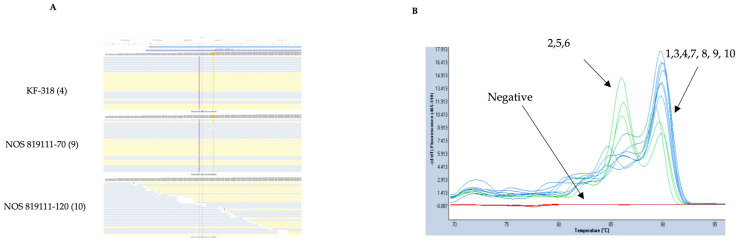
Read alignment of insertions in specific oat genotypes (**A**), the purple line show the insertion of 3b and orange line show the SNP in KF-318 and NOS 819111-70 but absent in NOS 819111-120. (**B**) HRM chromatogram of all 10 oat genotypes. Genotypes are represented as follows: (1) Symphony, (2) Delfin, (3) NOS 81920-15, (4) KF-318, (5) Mathilda, (6) WPB Oskar, (7) NOS 81937-11, (8) NOS 81950-13, (9) NOS 819111-70, and (10) NOS 819111-120. Selected insertion is located on chromosome 2A at position 456585644. Read alignment obtained through BWA-MEM mappers and genotype NOS 819111-120 used as example for remaining seven oat genotypes that exhibit similar alignment pattern as observed in NOS 819111-120. Alignment for all genotypes provided in Supplementary Figure S4.

For the selected insertion variants, the 3 bp insertion at position 456585644 of 2A showed a total depth coverage of 69 (127 reads) in KF-318 and 89 (163 reads) in NOS 819111-70. The genotype NOS 819111-120 has coverage of 311 (573 reads) for this region; read alignment confirmed the presence of the 3 bp insertion in both genotypes but not in NOS 819111-120 (Figure 5A). In fact, we did not observe this 3bp insertion in any of the genotypes except in KF-318 and NOS 819111-70 (Supplementary Figure S4). HRM analysis grouped genotypes KF-318, NOS 819111-70, and NOS 819111-120 together despite NOS 819111-120 lacking the 3 bp insertion (Figure 5B). Discrepancies between target capture data and validation results highlight potential issues in the applicability of myBaits technology in oat breeding. Several factors could contribute to these discrepancies, including biases in probe capture efficiency, high sequence variability, and the polyploid complexity of the oat genome. Additionally, the genetic diversity and mosaic nature of the oat genomes may pose challenges for effectively capturing and accurately calling variants. Hence, inadequate or uneven coverage, sequencing errors, and aligner inefficiencies may also play roles. Strategies to improve variant detection accuracy include, e.g., (i) the optimization of bait design and hybridization conditions specific to the oat genome, which could reduce biases and enhance capture efficiencies, (ii) the development of a specific bioinformatics pipeline, method, or tool to deal with target capture data generated from the oat genome, particularly considering the polyploidy complexity of the oat genome, high sequence variability, etc., (iii) implementing even more stringent quality-control measures during read-mapping and variant-calling processes, which can help identify and rectify errors, ensuring more reliable variant detection, and (iv) long-read sequencing technologies, which can provide more comprehensive coverage and accurate capture of variants, especially in complex and repetitive regions, reducing false positives.

## 4. Discussion

The findings of this study have broader implications for oat genomics research and marker development. The challenges and limitations observed with the current target-capture-sequencing approach demonstrate the need for continuous improvement in library preparation to capture the targeted regions in an unbiased manner, achieve an improvement in sequencing technologies, and develop innovative bioinformatics tools to handle the complexity of oat genomes effectively. Improved variant detection accuracy will directly impact the development of molecular markers, which are critical for breeding programs aimed at improving oat varieties. Ultimately, accurate and reliable markers will facilitate the selection of desirable traits, accelerate the breeding process, and improve crop yields, disease resistance, and stress tolerance. By addressing the limitations identified in this study and implementing the proposed strategies, oat researchers can develop more precise and effective markers, contributing to the overall advancement of oat breeding and agricultural productivity. Moreover, the insights gained from this study can be applied to other polyploid and complex plant genomes, broadening the impact of this research beyond oats. As sequencing technologies and bioinformatics tools continue to evolve, the potential for groundbreaking discoveries in plant genomics and breeding will expand.

The process of variant discovery in oat genomics involves two primary stages: read alignment and variant calling. A plethora of tools exist for each stage; hence, the use of different aligners and variant callers may be crucial to evaluating and confirming the effectiveness of certain sequencing technologies. Accordingly, different aligners and variant callers were employed in this study. Many plant studies involve high levels of genetic diversity and, in some cases, incorporate distantly related varieties and wild relatives [17,26]. Neither of these conditions is common in human studies, and as such, pipelines designed and evaluated on humans may perform differently than expected [27,28]. Therefore, in this study, we employed three different aligners commonly used in plant genomic studies, namely BWA-MEM, Bowtie2, and NGSEP, to map the Illumina paired-end read target-capture datasets from 10 oat genotypes. Our results demonstrated that BWA-MEM consistently exhibited high mapping efficiency across all genotypes, with percentages ranging from 98.98% to 99.84%. Earlier, Yan et al., 2021, also identified that BWA-MEM has a higher mapping rate than Bowtie 2 when they evaluated these two mappers using large plant genome resequencing data [16]. However, BWA-MEM’s increased sensitivity may come at a cost in that, as the number of SNPs or the size of the INDELs per read increased, the false positive rate also became slightly higher than that of Bowtie and NGSEP. Although Bowtie2 and NGSEP also showed comparable mapping efficiencies, slight variations were observed across genotypes. These findings suggest that the use of different aligners is the way forward in oat genomics analyses due to its mosaic genome and complexity. Similar results were obtained by Schilbert et al., 2020, when they compared different mappers using plant NGS data [17]. Neither NGSEP nor Bowtie2 was able to align as many reads for any genotype when compared with the BWA-MEM mapper. In this study, we chose not to alter the default setting mostly because the mapping percentage was already high and there was no obvious parameter, such as the number of mismatches allowed or the fragment size. Moreover, many program users, especially non-experts in bioinformatics, may retain the default settings of programs. The results of this study are also in line with other studies in which similar results were previously reported, suggesting that the BWA-MEM mapping tool had a higher mapping rate [16,17]. However, we recommend using different mappers for variant discovery to lower the false discovery rate. Our study also has strengths compared to other studies in that different mappers were employed when utilizing real data obtained through the target capture sequencing of several oat genotypes, rather than testing using simulated sequence data.

The next step in the bioinformatic analysis pipeline is variant calling, and we performed variant calling using five variant callers. These variant callers include the GATK Haplotype Caller (GATK-HC), FreeBayes, DeepVariant, the NGSEP variant caller, and SAMtools-mpileup. GATK-HC detected different variant counts, ranging from 3816 for Symphony to 4411 for NOS 819111-120. In contrast, SAMtools-mpileup identified fewer variants, ranging from 753 for Symphony to 2820 for NOS 819111-120. GATK-HC detected many variants compared to SAMtools-mpileup, which resulted in a very low recall of variants. The reason could be that GATK-HC performs local assembly to identify the haplotypes, whereas SAMtools-mpileup only utilizes read alignments. Plant genomes, in general, are rich in repetitive sequences that are difficult to assemble correctly using short reads. Therefore, the local assembly strategy employed via GATK-HC might identify true variants, but on the other hand, it might also generate false positive variants, especially INDELs. FreeBayes demonstrated a broad spectrum of variants across genotypes, with counts ranging from 544 for NOS 81937-11 to 4338 for NOS 81920-15. DeepVariant exhibited variant counts ranging from 513 for NOS 81937-11 to 2185 for Symphony. Lastly, NGSEP Variant consistently detected many variants across genotypes, ranging from 3223 for Symphony to 4325 for NOS 819111-120. These results highlight that different variant callers detect different numbers of variants; hence, it is advisable to use different variant callers and then select variants that are common among callers. We chose this strategy in our study. We found that all the variants called via DeepVariant were also detected via at least one other variant caller. The DeepVariant method relies on a convolutional neural network model, and such advanced machine-learning techniques hold significant promise for the future evolution of bioinformatic software, particularly in variant-calling applications [29]. Hence, if someone wanted to use only a variant caller, then DeepVariant could be a better choice. Studies revealed that the DeepVariant method can detect variants utilizing next-generation sequencing (NGS) data with accuracy [30,31,32]. However, it is always better to use combinations of variant callers and then choose the variants detected via the number of variant callers. This strategy has been employed in various studies previously [33,34].

The main finding of our study is that the target capture methodology using short-read sequencing devised by Daicel Arbor Biosciences is not applicable in oat genome research to identify variants with reliability. We reached this conclusion in the validation step when we used the variants identified via all five variant callers employed in this study. Among the variants called, we selected variants detected via all variant callers in two oat genotypes, i.e., KF-318 and NOS 819111-70, but which were absent in the remaining genotypes for validation purposes. In the case of the selected SNPs, the Sanger sequencing of the target region contradicted the target capture data even though the coverage of selected variants in the target capture data was very high. Similarly, the validation of deletion and insertion variants also presented challenges, suggesting limitations in the reliability of myBaits target capture sequencing for variant detection in the oat genome. To further investigate these discrepancies, we conducted high-resolution melting (HRM) curve analysis, which grouped the ten oat genotypes into various clusters, indicating the presence of variations in the targeted regions. Interestingly, some genotypes that were not expected to cluster together were observed to do so. While Sanger sequencing could have been employed to verify and elucidate these variations, we refrained from this approach due to the contradictory results observed in the target capture data for SNPs when Sanger sequencing was performed. Challenges primarily revolve around the reliability of target capture data in ensuring precise variant-calling accuracy using these target capture data. Given that the myBaits technology involves the targeted sequencing of specific genomic regions, the accurate capture of variants within these regions is, of course, of vital significance. Challenges may surface in accurately capturing variants, especially in regions with high sequence variability or complexity. Inadequate coverage via myBaits probes or biases in capture efficiency could lead to incomplete variant calling or inaccuracies in variant identification. In our situation, we have seen sufficient coverage; hence, biases in capture efficiency could be the cause. These challenges arise due to the complexity of oat genomes, which contain large repetitive regions and polyploidy, making efficient bait design and target capture difficult. Additionally, the genetic diversity and mosaic nature of the oat genome might pose challenges in ensuring that baits effectively capture target sequences. The methodology of custom bait design and synthesis can also be a reason, especially for large genomes such as oats. We suggest that bait design and hybridization conditions should be optimized for oats, and this may require extensive experimentation to ensure reliability.

While our study highlights limitations in the reliability of target-capture sequencing using short-read sequencing for variant detection in the oat genome, long-read sequencing can be useful in this context. The myBaits technology using long-read sequencing instead of short-read sequencing could be a better option for the reliable detection of variants. However, this needs to be tested in the case of a complex and polyploid genome, such as the oat genome. Long-read sequencing offers a promising strategy to mitigate the challenges encountered in target-capture sequencing using short reads and to avoid false positive results [35]. By generating longer sequencing reads, long-read sequencing technologies can overcome some of the challenges associated with short-read sequencing, such as accurately capturing variants in regions with high sequence variability or complexity. Additionally, long-read sequencing enables a more comprehensive characterization of genetic variation, including large structural variants and complex rearrangements, which may be missed or inaccurately identified via short-read sequencing [36]. However, this needs to be validated for the oat genome to conclude that log-read sequencing is the solution for the shortcoming of the myBaits target-capture technology in the reliable detection of variants.

It is true that the myBaits technology offers substantial opportunities for plant researchers by enabling the targeted sequencing of specific regions of interest; researchers can concentrate on genomic regions associated with the traits of interest or genetic variation [13,37]. This approach enhances variant-calling efficiency by reducing the volume of non-targeted sequencing data that require processing, potentially alleviating computational burdens and associated costs [8,11,12,15]. Furthermore, the flexibility in bait design provided via the myBaits technology empowers researchers to tailor sequencing experiments to align with specific research goals or genomic regions of interest. However, our study revealed limitations regarding the suitability of the myBaits target capture technology for marker development in oat breeding. Despite its advantages, the technology may not yet meet the stringent requirements for marker development in oat breeding programs.

To address the limitations of myBaits target-capture sequencing observed in this study and advance research in oat genomics and marker development, we propose the following recommendations for future research directions. (i) The further refinement of myBaits protocols is essential, as the current protocol does not seem to work reliably for variant detection in oats. This may involve fine-tuning the probe design, optimizing the hybridization conditions, and enhancing the coverage depth to improve the accuracy and efficiency of variant calling. Moreover, different hybridization conditions should be explored, including temperature, duration, and buffer composition, to optimize the efficiency of target capture. Fine-tuning these parameters can improve the specificity and sensitivity of the hybridization process. (ii) Incorporating long-read sequencing technologies, such as PacBio or Oxford Nanopore, alongside myBaits target capture can be a useful strategy to overcome the challenges posed due to the complex oat genome. Long reads can provide valuable information for resolving repetitive regions and structural variations and address the bioinformatic challenges associated with short reads. However, the feasibility and effectiveness of this approach need further investigation in the context of oats and myBaits target capture. (iii) Alternative targeted sequencing methods, such as amplicon-based sequencing, may offer advantages for marker development to facilitate oat breeding. Amplicon sequencing can provide a targeted approach while avoiding some of the challenges associated with myBaits, such as probe design limitations and capture biases. (iv) The development of specific bioinformatics pipelines or tools tailored to analyzing target capture data generated via myBaits from the oat genome is crucial. These tools should account for the unique challenges posed due to the polyploid nature of oats, high sequence variability, and the presence of repetitive regions. By developing specialized computational methods, researchers can more effectively process and interpret target-capture data, leading to more reliable variant detection and downstream applications. By implementing these recommendations and leveraging recent advancements in oat genomics, researchers can overcome the limitations of myBaits target capture sequencing and unlock the full potential of targeted sequencing technologies for oat breeding.

## 5. Conclusions

In conclusion, our study sheds light on the use of myBaits technology for variant detection in oat genomics, highlighting both its potential and limitations. While the myBaits technology offers an efficient approach to targeted sequencing and variant detection in other crops, our findings highlight the need for a cautious interpretation of results, particularly concerning complex polyploid genomes such as oats. The discrepancies observed in variant validation highlight challenges in ensuring the accuracy and reliability of variant calling using the myBaits technology. Moving forward, it is imperative to refine and optimize myBaits protocols to enhance their efficacy and reliability in oat genomics research. Our study emphasizes the importance of methodological considerations and validation strategies in oat genomics research, paving the way for further advancements in this important field.

## Figures and Tables

**Table 1:** Read statistics and mapping efficiency of BWA-Mem, Bowtie 2, and NGSEP aligners.

Genotype	QC_Passed Reads	BWA-MeM		Bowtie 2		NGSEP	
		Mapped Reads	Efficiency %	Mapped Reads	Efficiency %	Mapped Reads	Efficiency %
Symphony	47227270	47100831	99.73	44962680	95.20	44849377	94.97
Delfin	34580186	34228086	98.98	33489584	96.85	32994684	95.42
NOS 81920-15	44241902	44173144	99.84	42755214	96.64	42357197	95.74
KF-318	31651718	31332372	98.99	30625580	96.76	30346085	95.88
Mathilda	44142112	44037777	99.76	42811726	96.99	42541960	96.38
WPB Oskar	29818026	29760040	99.81	28901044	96.92	28734141	96.37
NOS 81937-11	43716198	43270628	98.98	42451392	97.11	41550060	95.05
NOS 81950-13	43716198	43270628	98.98	42451392	97.11	41550060	95.05
NOS 819111-70	37052870	36874093	99.52	34308912	92.59	33367962	90.06
NOS 819111-120	48525636	48382873	99.71	45956124	94.70	45713575	94.21

**Table 2:** Numbers of variants identified in 10 oat genotypes using different mappers and variant callers.

Mappers	Variant Callers	Genotypes									
		Symphony	Delfin	NOS 81920-15	KF-318	Mathilda	WPB Oskar	NOS 81937-11	NOS 81950-13	NOS 819111-70	NOS 819111-120
BWA-MEM	GATK Haplotype Caller	3816	4405	4043	3874	4406	3903	4414	4255	3906	4411
	FreeBayes	4310	1034	4338	1924	1099	2329	544	2285	2099	1094
	DeepVariant	2185	994	2159	1126	1070	843	513	509	976	1051
NGSEP	NGSEP Variant caller	3223	4092	4563	3832	4029	3653	4123	4111	3671	4325
Bowtie 2	SAMtools mpileup	753	2815	957	1989	2803	2818	2406	2399	1975	2820

## Data Availability

All the raw sequencing data was deposited in NCBI with accession number 1095189, and they are accessible via https://www.ncbi.nlm.nih.gov/sra, accessed on 10 April 2024.

## Supplementary Materials

The following supporting information can be downloaded at https://www.mdpi.com/article/10.3390/genes15060700/s1, Figure S1: Read alignment of target capture sequencing data of oat genotypes obtained through BWAMEM. The SNP located on chromosome 2A at position 456055130 is the targeted Variant. Oat genotypes are arranged as follows: KF-318, NOS 819111-120, NOS 819111-70, NOS 81920-15, NOS 81937-11, NOS 81950-13, Delfin, Mathilda, WPB Oskar, Symphony; Figure S2: Read alignment of target capture sequencing data of oat genotypes obtained through BWAMEM. The SNP located on chromosome 2A at position 455932982 is the targeted Variant. Oat genotypes are arranged as follows: KF-318, NOS 819111-120, NOS 819111-70, NOS 81937-11, NOS 81950-13, Delfin, Mathilda and WPB Oskar; Figure S3: Read alignment of target capture sequencing data of oat genotypes obtained through BWAMEM. The deletion located on chromosome 2A at position 453603957 is the targeted Variant. Oat genotypes are arranged as follows: KF-318, NOS 819111-120, NOS 819111-70, NOS 81920-15, NOS 81937-11, NOS 81950-13, Delfin, Mathilda, WPB Oskar, Symphony; Figure S4: Read alignment of target capture sequencing data of oat genotypes obtained through BWAMEM. The insertion located on chromosome 2A at position 456585644 is the targeted Variant. Oat genotypes are arranged as follows: KF-318, NOS 819111-120, NOS 819111-70, NOS 81920-15, NOS 81937-11, NOS 81950-13, Delfin, Mathilda, WPB Oskar, Symphony; Table S1: Primers flanking the variants for validation study.
