# Supplementary material for: Assessing myBaits Target Capture Sequencing Methodology Using Short-Read Sequencing for Variant Detection in Oat Genomics and Breeding
Source: Genes (Basel). 2024 May 27;15(6):700. doi: 10.3390/genes15060700 (PMC11203172; doi:10.3390/genes15060700)
Supplement: Supplementary file 1 [file genes-15-00700-s001.zip › genes-3011501-supplementary.pdf]

Supplementary files

1. Read alignment of target capture sequencing data of oat genotypes obtained through BWA-MEM. The SNP located on chromosome 2A at position 456055130 is the targeted Variant. Oat genotypes are arranged as follows: KF-318, NOS 819111-120, NOS 819111-70, NOS 81920-15, NOS 81937-11 NOS 81950-13,Delfin, Mathilda, WPB Oskar, Symphony. (fig. 1, Page 2)

2. Read alignment of target capture sequencing data of oat genotypes obtained through BWA-MEM. The SNP located on chromosome 2A at position 455932982 is the targeted Variant. Oat genotypes are arranged as follows: KF-318, NOS 819111-120, NOS 819111-70, NOS 81937-11 NOS 81950-13,Delfin, Mathilda and WPB Oskar. (fig. 2,Page 3)

3. Read alignment of target capture sequencing data of oat genotypes obtained through BWA-MEM. The deletion located on chromosome 2A at position 453603957 is the targeted Variant. Oat genotypes are arranged as follows: KF-318, NOS 819111-120, NOS 819111-70, NOS 81920-15, NOS 81937-11 NOS 81950-13,Delfin, Mathilda, WPB Oskar, Symphony. (fig. 3, Page 4)

4. Read alignment of target capture sequencing data of oat genotypes obtained through BWA-MEM. The insertion located on chromosome 2A at position 456585644 is the targeted Variant. Oat genotypes are arranged as follows: KF-318, NOS 819111-120, NOS 819111-70, NOS 81920-15, NOS 81937-11 NOS 81950-13,Delfin, Mathilda, WPB Oskar, Symphony. (fig. 4, Page 5)

5. Supplementary Table 1: Primers flanking the variants for validation study (Page 6)

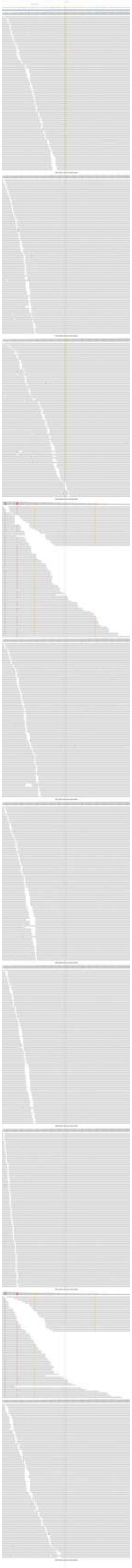

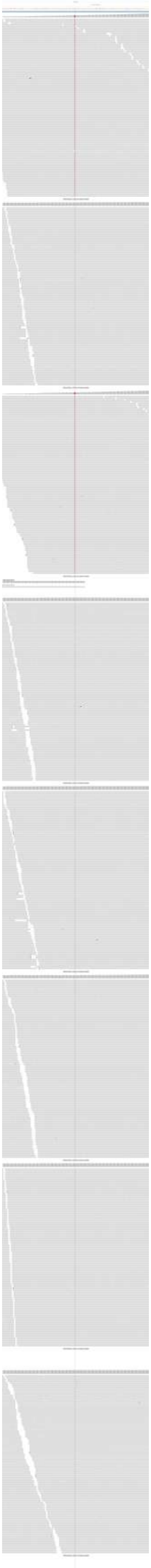

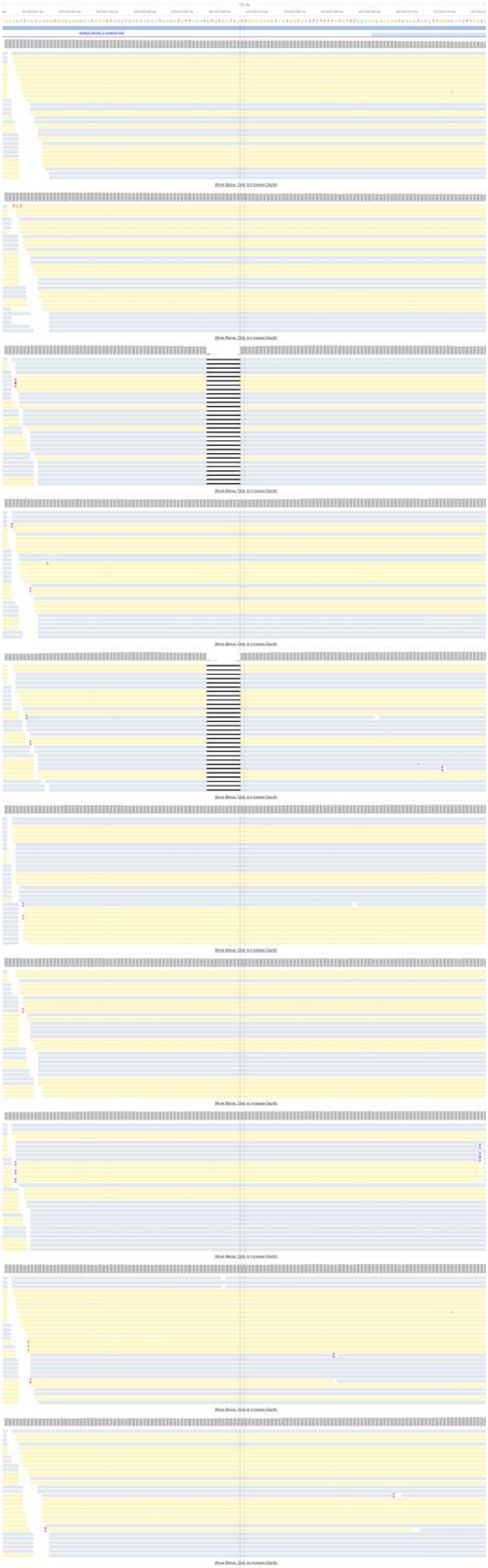

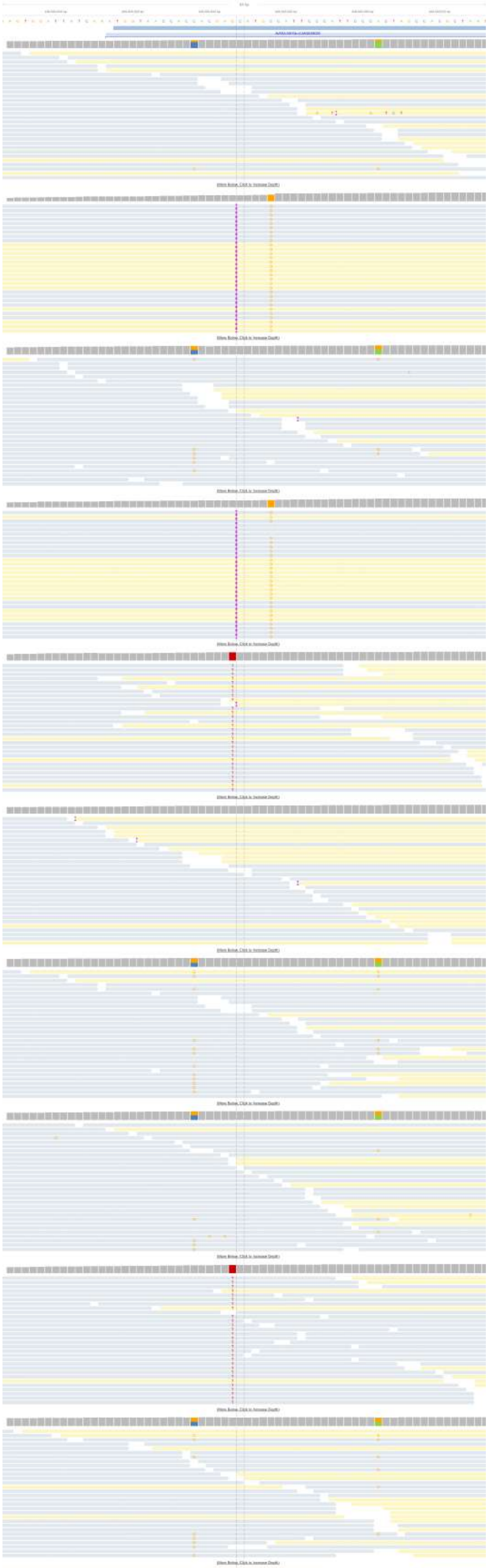

Supplementary Table 1: Primers flanking the variants for validation study

Name	Type	Gene	Sequence (5’-3’)	Validation method	PCR product (bp)
2A_456055130	SNP	AVESA.00010b.r2.2AG0258170	456055130_F-GTGCTCTGCCCCCTGTATTC<br>456055130_R-CCATCGTCGTTGTCCACCAT	Sanger	500
2A_455932982	SNP	AVESA.00010b.r2.2AG0258110	455932982_F-CCTCTGTTTGCTTCTCCACCA<br>455932982_R-AACACCTCCGGTTCAGCAAG	Sanger	500
9bp_453603957	Deletion	AVESA.00010b.r2.2AG0257420	7420_F-AACATACTGTCGTGGTCGGC<br>7420_R-ACCTTCGCAGCAATAGCCAA	HRM	169
3bp_456585644	Insertion	AVESA.00010b.r2.2AG0258320	258320_F-CCGTCAAGTGGATTATCAAATGGT<br>258320_R-GTGGAGGTTGCCGACGATC	HRM	181
